# Microarray study reveals that HIV-1 induces rapid type-I interferon-dependent p53 mRNA up-regulation in human primary CD4^+ ^T cells

**DOI:** 10.1186/1742-4690-6-5

**Published:** 2009-01-15

**Authors:** Michaël Imbeault, Michel Ouellet, Michel J Tremblay

**Affiliations:** 1 Centre de Recherche en Infectiologie, Centre Hospitalier de l'Université Laval, and Faculté de Médecine, Université Laval, Québec, Canada

## Abstract

**Background:**

Infection with HIV-1 has been shown to alter expression of a large array of host cell genes. However, previous studies aimed at investigating the putative HIV-1-induced modulation of host gene expression have been mostly performed in established human cell lines. To better approximate natural conditions, we monitored gene expression changes in a cell population highly enriched in human primary CD4^+ ^T lymphocytes exposed to HIV-1 using commercial oligonucleotide microarrays from Affymetrix.

**Results:**

We report here that HIV-1 influences expression of genes related to many important biological processes such as DNA repair, cellular cycle, RNA metabolism and apoptosis. Notably, expression of the p53 tumor suppressor and genes involved in p53 homeostasis such as GADD34 were up-regulated by HIV-1 at the mRNA level. This observation is distinct from the previously reported p53 phosphorylation and stabilization at the protein level, which precedes HIV-1-induced apoptosis. We present evidence that the HIV-1-mediated increase in p53 gene expression is associated with virus-mediated induction of type-I interferon (i.e. IFN-α and IFN-β).

**Conclusion:**

These observations have important implications for our understanding of HIV-1 pathogenesis, particularly in respect to the virus-induced depletion of CD4^+ ^T cells.

## Background

Infection by human immunodeficiency virus type-1 (HIV-1) is characterized by a progressive degradation of the human immune system, a condition better known as the acquired immunodeficiency syndrome (AIDS). The process by which this breakdown occurs has been the subject of intense research in the past few years. It appears that HIV-1 causes a slow but progressive death of CD4^+ ^T lymphocytes, which are key players of the immune system that coordinate the humoral and cellular responses. However, the exact mechanism(s) leading to such a dramatic depletion of CD4^+ ^T cells *in vivo *is not well understood, although it has been proposed that this phenomenon is multifactorial [1]. It has been suggested that apoptosis or programmed cell death plays a dominant role in the observed HIV-1-mediated CD4^+ ^T cell depletion. Recent studies have identified numerous viral components that can induce apoptosis via different pathways. Indeed, the viral proteins Tat [2], Nef [3], Vpr [4] and gp120 [5] can all elicit apoptosis in CD4^+ ^T lymphocytes, at least under *in vitro *conditions. Even if the actual relevance and *in vivo *impact of these studies remain to be established, it is clear that HIV-1 interactions with its host are complex and multifaceted.

New technologies are rapidly expanding our analytical power. Among the technical innovations developed in the past few years, cDNA and oligonucleotide microarrays have revolutionized the way we look at and understand gene expression, allowing the rapid quantification of thousands of genes at once in a given cell population. Recently, microarrays have been used by different groups to determine the effects of whole HIV-1 particles or single viral proteins (e.g. Tat [6] and Nef [7]) on CD4^+ ^T lymphoid cell lines, monocytoid cell lines, primary astrocytes [8-11], primary macrophages [12] and jejunal biopsies [13]. A comprehensive review of the 34 studies involving HIV-1 and microarrays in the 2000–2006 period is available [14]. These studies yielded important data on HIV-1-mediated effects on gene expression, providing new insights into the intricate interactions occurring during infection. Nevertheless, there is still a paucity of data regarding the modifications in gene expression profiles induced by HIV-1 in human primary CD4^+ ^T lymphocytes, a cell type considered as a major target for HIV-1. Only two recent studies have performed gene expression analyses in this major cell reservoir for HIV-1. A first analysis has compared the genetic profiles between viremic and aviremic HIV-1 positive individuals in a population of resting CD4^+ ^T cells [15]. More recently, an elegant study by Audigé and colleagues has examined the impact of HIV-1 infection on resting CD4^+ ^T cells extracted from *ex vivo *tonsils [16]. Consequently, we felt it was crucial to provide additional information on possible changes in early gene expression following exposure of activated human primary CD4^+ ^T lymphocytes to HIV-1 particles. The rationale for such a study is provided by the idea that cell lines, which have often been preferred over primary cells for microarray studies involving HIV-1, are either cancerous or transformed by viral proteins, and can thus harbour numerous defects in multiple pathways compared to primary cells, notably in their apoptosis-related metabolism, cell cycle and DNA repair functions. We thus decided to run a small-scale study focusing on early transcriptional events following HIV-1 infection in activated primary CD4^+ ^T cells isolated from peripheral blood.

We considered that focusing on early events following exposure to HIV-1 had the potential to yield the most interesting results as cell signalling events and gene expression changes can occur in just a few hours. Our goal was to identify a small set of regulated genes that could be confirmed by quantitative real-time PCR (qRT-PCR) and western blot analyses. Additionally, as our laboratory has extensively characterized the effect of ICAM-1 incorporation in the virus lipid bilayer [17-23], we investigated whether the presence of host-derived ICAM-1 onto HIV-1 would influence the virus-mediated changes in the transcriptional profiles. In the current work, results depicting the early gene modulation initiated by HIV-1 in a cell population highly enriched in CD4^+ ^T lymphocytes using Affymetrix microarray technology are presented.

## Methods

### Cell culture

Peripheral blood was obtained from normal healthy donors and peripheral blood mononuclear cells (PBMCs) were prepared by centrifugation on a Ficoll-Hypaque density gradient. Next, a cell population highly enriched in CD4^+ ^T cells was isolated through the use of the human CD4^+ ^T Cell Isolation Kit II™ (Miltenyi Biotec, Auburn, CA) according to the manufacturer's instructions. Some experiments have also been performed with another negative selection kit designed for the purification of human CD4^+ ^T cells (StemCell Technologies Inc., Vancouver, BC). The purity of the negatively selected cell population was estimated by quantifying the percentage of CD4-expressing cells. Next, cells were cultured at a concentration of 2 × 10^6^/ml in complete RPMI-1640 medium (Invitrogen, Burlington, ON) supplemented with 10% fetal bovine serum (FBS) (Atlanta Biologicals, Norcross, GA), L-glutamine (2 mM), penicillin G (100 U/ml), streptomycin (100 μg/ml), phytohemagglutinin-L (1 μg/ml) and recombinant human IL-2 (30 U/ml) for 3 days at 37°C under a 5% CO_2 _atmosphere prior to virus infection. Human embryonic kidney 293T cells and .HEK-Blue™ IFN-α/β cells (InvivoGen, San Diego, CA) were maintained in Dulbecco's modified Eagle medium (Invitrogen) supplemented with 10% FBS, glutamine (2 mM), penicillin G (100 U/ml) and streptomycin (100 mg/ml). Culture media used for .HEK-Blue™ IFN-α/β cells was supplemented with 30 μg/ml of blasticidin and 100 μg/ml of Zeocin.

### Production of virus stocks

Isogenic virus particles differing only by the absence or the presence of host-derived ICAM-1 proteins on their outer membranes were produced by calcium phosphate transfection in 293T cells using a commercial calcium phosphate co-precipitation kit according to the manufacturer's instructions (CalPhos Mammalian Transfection kit, Clontech Laboratories Inc., Palo Alto, CA). Briefly, parental 293T cells were transiently co-transfected with pNL4-3 (an infectious X4-tropic infectious molecular clone of HIV-1) [24] to produce viruses lacking host ICAM-1 (called NL4-3 wt). Moreover, 293T cells engineered to constitutively express a high level of ICAM-1 (i.e. 293T-ICAM-1) [25] were similarly transfected with pNL4-3 to produce ICAM-1-bearing viruses (called NL4-3 ICAM-1^+^). The NL4-3 vector was obtained from the NIH AIDS Repository Reagent Program (Germantown, MD). In some experiments, the percentage of cells productively infected with HIV-1 was estimated through the use of fully competent GFP-encoding viruses, which were produced by transfecting 293T and 293T-ICAM-1 cells with the infectious molecular clone NLENG1-IRES (NL4-3-based vector) (a generous gift from D.N. Levy, New York University, NY) [26]. Cell-free supernatants from such transiently transfected cells were filtered through a 0.22-μm-pore-size cellulose acetate membrane (Millipore, Bedford, MA). To eliminate free p24, cell-free supernatants were treated using Centricon^® ^Plus-20 Biomax-100 filter devices (Millipore Corporation) or ultracentrifugation. Finally, samples were aliquoted before storage at -85°C. A p24 antibody capture assay developed in our laboratory was used to normalize the p24 content in all viral preparations [27]. All virus preparations underwent a single freeze-thaw cycle before initiation of infection studies.

### Flow cytometry

Flow cytometry analyses were performed with a total of 10^6 ^cells that were incubated with 100 μl of PBS (pH 7.4) containing a saturating amount of a monoclonal anti-CD4 or anti-CD14 antibody for 30 min on ice. Thereafter, cells were treated with a pool of human serum for 30 min at 4°C and then washed with cold PBS, in order to block Fc receptors and non-specific sites. The cells were then labelled for 30 min at 4°C with 100 μl of a saturating amount of FITC-conjugated goat anti-mouse immunoglobulin G (Caltag, Invitrogen). Finally, cells were washed, fixed in 2% paraformaldehyde for 30 min and analyzed on a cytofluorometer (EPICS XL, Coulter Corp., Miami, FL).

### Microarray experiments

A cell population highly enriched in CD4^+ ^T cells was either left unexposed or exposed to NL4-3 particles either lacking (NL4-3 wt) or bearing host-derived ICAM-1 (NL4-3 ICAM-1+) for 8 and 24 h at 37°C. A virus input of 10 ng of p24 per 1 × 10^5 ^target cells was used in all studies. RNA samples from five healthy donors were pooled together to minimize experimental variations. Cell pellets were frozen at -80°C until isolation of total mRNA was performed using the RNeasy kit according to the manufacturer's protocol (Qiagen, Valencia, CA). All samples were processed at the same time and using the same kit. The RNA quality was controlled by electrophoresis on a denaturing gel as specified in the Affymetrix's protocol. Gene expression profiles were analyzed using commercial oligonucleotide microarrays (HGU95Av2 GeneChips, Affymetrix, Santa Clara, CA), which contain probe sets representing 12,627 transcripts. A total of six microarrays were used, i.e. mock-infected, infected with NL4-3, or infected with NL4-3 ICAM-1+ at 8 and 24 h post-infection. Affymetrix standard protocols were followed throughout these experiments. Data were globally normalized (target: 1000) and present calls were determined using MAS 5.0 (Microarray Suite v5.0, Affymetrix, Santa Clara, CA). Results were analyzed using GeneSpring 6.0 (Agilent Technologies, Santa Clara, CA). Signal intensity was normalized for each microarray and genes with a signal below 100 were ignored. Fold changes of two times the control and higher were considered as significant. GO overrepresentation analysis was performed with the GO Tree Machine software  using the "interesting gene list vs reference gene list" setting against the affy_HG_U95AV2 reference list.

### qRT-PCR analysis

The expression level of some specific transcripts was determined using a Rotor-Gene system (Corbett Life Science, Sydney, Australia). Total RNA was isolated using the Qiagen RNA extraction kit and then digested with deoxyribonuclease to remove any contaminating genomic DNA. RNA was reverse-transcribed using AMV reverse transcriptase (Promega). We then proceeded to qRT-PCR quantification of transcripts using Taq polymerase (AmpliTaq Gold^® ^PCR Master Mix, Applied Biosystems) and Sybr Green detection. Normalization on 18S mRNA levels was performed to obtain final expression values. A standard curve was drawn for each gene of interest by serial dilutions of a pool of RNA. The sequence of primers we used is presented in Table 1.

**Table 1:** Primers sequences used for qRT-PCR analysis

**Primer name**	**Sequence**
**p53 sense**	5'-ACAGCACATGACGGAGGTTG-3'
**p53 antisense**	5'-CCCAGGACAGGCACAAACAC-3'
**ribosomal 18S sense**	5'-TGTTCAAAGCAGGCCCGAG-3'
**ribosomal 18S antisense**	5'-CGGAACTACGACGGTATCTGATC-3'
**GADD34 sense**	5'-AACCTCTACTTCTGCCTTGTCT-3'
**GADD34 antisense**	5'-CGCCTCTCCTGAACGATACTC-3'
**TNFRSF25 sense**	5'-GGAGAACCACCATAATTC-3'
**TNFRSF25 antisense**	5'-TCTTCCTATTCCTGAACC-3'

### Western blots

A cell population highly enriched in CD4^+ ^T lymphocytes was either left unexposed or exposed to viruses lacking host-derived ICAM-1 (i.e. NL4-3 wt) for 24 and 48 h at 37°C. Thereafter, total cell extracts were heated at 100°C for 10 min in 1× sample buffer (62 mM Tris-HCl [pH 6.8], 2% SDS, 5% β-mercaptoethanol, 9% glycerol and 0.002% bromophenol blue) containing 1 mM PMSF. The samples were then electrophoresed on a 7.5 to 20% gradient sodium dodecyl sulfate-polyacrylamide gel and transferred to Immobilon polyvinylidene difluoride membranes (Millipore, Bedford, MA). Immunoblotting was performed using antibodies specific for p53 (clone DO-1, Santa Cruz Biotechnology, Santa Cruz, CA), GADD34 (goat polyclonal antibody, Serotec, Raleigh, NC), TNFRSF25 (rabbit polyclonal antibody DR3 Ab-2, Neomarkers, Fremont, CA) and β-actin (mouse monoclonal antibody, clone C-2, Santa Cruz Biotechnology). Membranes were labelled with horseradish peroxidase-conjugated secondary anti-rabbit or anti-mouse antibodies (Jackson ImmunoResearch, Mississauga, ON) at a 1:20,000 and 1:10,000 dilution, respectively. Signals were revealed using the ECL™ Western blotting detection reagent (Amersham, Piscataway, NJ). Densitometry analysis was performed using the freely available image analysis ImageJ software .

### Measurements of IFN-α/β and blocking experiments

Levels of interferon-α (IFN-α) and IFN-β in cell-free supernatants from the studied cell populations highly enriched in CD4^+ ^T cells either unexposed or exposed to virus stocks were determined through the use of .HEK-Blue™ IFN-α/β cells according to the manufacturer's protocol (InvivoGen, San Diego, CA). Supernatants were collected at 1, 2, 4 and 6 h following virus exposure. Virus was added in a reverse time course and all supernatants were harvested simultaneously. A standard curve of IFN-α ranging from 1 to 1,000 Units/ml was used. In the neutralizing experiments, antibodies that can inhibit human IFN-α (MMHA-2 from PBL Interferon Source, Piscataway, NJ or ab9660 from Abcam, Cambridge, MA) and IFN-β (ab9662 from Abcam) were mixed together at equal concentrations (i.e. 1 μg/ml) and added simultaneously with viruses. Appropriate isotype-matched control antibodies were also used. After an incubation period of 24 h, total RNA was extracted and p53 and 18S mRNA levels were quantified by qRT-PCR as previously described.

### Statistical analysis

Means were compared using the Student's test. P values of less than 0.05 were considered statistically significant. Microsoft Office Excel 2007 software was used for all statistical analyses.

## Results

### Characterization of the studied cell population

**Figure 1:**
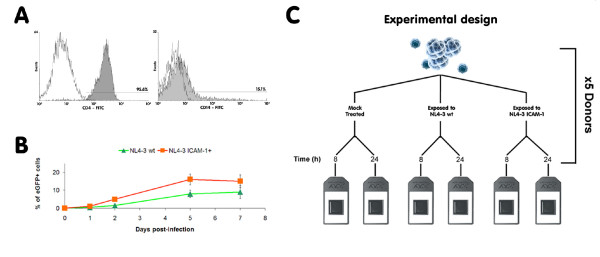
**Characterization of the studied cell population and overview of the experimental design**. (A) PBMCs were subjected to magnetic-based CD4^+ ^T cell negative selection. Percentages of CD4^+ ^(left panel) and CD14^+ ^cells (right panel) in the enriched population were estimated by flow cytometry immediately after selection. The right panel represents a worst-case scenario for contaminating CD14^+ ^cells. (B) The studied cell population highly enriched in CD4^+ ^T cells were infected with fully infectious eGFP-encoding viruses either lacking (NL4-3 wt) or bearing host-derived ICAM-1 (NL4-3 ICAM-1+) and the number of cells productively infected with HIV-1 (i.e. eGFP-positive) was estimated by flow cytometry at the listed days following infection. (C) A schematic representation of the experimental design used for the microarray studies is shown.

It is known that experiments involving human primary cells are more difficult to perform than comparable studies using established cell lines, as one has to account for the inherent variability between donors, such as the state of cell activation and homogeneity of the isolated population, notwithstanding differences in genetic background. The purity of the studied CD4^+ ^T cell population isolated from PBMCs through a negative magnetic selection procedure was assessed by flow cytometry. The two commercial isolation kits used to purify human CD4^+ ^T lymphocytes (i.e. Miltenyi Biotec and StemCell Technologies Inc.) routinely yielded a degree of purity greater than 96%. However, flow cytometry analyses revealed the presence of cells positive for both CD4 and CD14 in a proportion ranging from 5 up to 15% in some rare cases (Fig. 1A), thus suggesting a variable but reproducible contamination with cells of monocytic lineage. To reflect the fact that the studied population of human primary cells was not made exclusively of CD4^+ ^T cells, we will refer to it as a cell population highly enriched in CD4^+ ^T cells.

### HIV-1 infection rate is low in the studied cell population

We next considered that it was crucial to estimate virus infection rates in our samples in order to accurately interpret results we would obtain from the microarray data. To this end, recombinant reporter virions were used to quantify the percentage of cells productively infected with HIV-1 at 1, 2, 5 and 7 days post-infection. Fully competent eGFP-encoding virions were produced upon transient transfection of parental 293T cells (to produce viruses lacking ICAM-1) and 293T-ICAM-1 (to produce ICAM-1-bearing virions) with the NLENG1-IRES vector. This infectious molecular clone of HIV-1 contains an eGFP-IRES-Nef construct in place of the Nef open reading frame within the backbone of NL4-3 [26]. Consequently, eGFP is expressed along with early genes, allowing for a rapid and precise quantification of the percentage of cells productively infected with HIV-1. Moreover, viruses produced by the NLENG1-IRES vector are fully infectious and express all viral genes, unlike previously described HIV-1 reporter constructs that are deficient in *nef*, *vpr *and/or *env*. Following exposure of the isolated cell population highly enriched in CD4^+ ^T cells to the viral input used (i.e. 10 ng of p24 per 1 × 10^5 ^cells), we found that on average less than 10% of cells are expressing the virus-encoded reporter protein at 5 days post-infection when infection was allowed to proceed with viruses lacking host-derived ICAM-1 (i.e. NL4-3 wt) (Fig. 1B). As expected, the number of cells that are productively infected is enhanced when infection is performed with isogenic ICAM-1-bearing HIV-1 particles (i.e. NL4-3 ICAM-1+) resulting in more than 15% eGFP^+ ^cells at five days post-infection. This observation is consistent with the reported increase in p24 production following infection with ICAM-1-bearing viruses [19]. The viral infection rates that are seen following exposure to HIV-1 either lacking or bearing host-derived ICAM-1 are very low at the two time points studied in the microarray experiment (i.e. 8 and 24 h post-infection). Next, comparative analyses were made to evaluate the permissiveness of human CD4^+ ^T lymphoid cells to the studied reporter viruses. To this end, Jurkat cells were exposed to a similar input of eGFP-encoding virions and the percentage of positive cells was monitored by flow cytometry. In sharp contrast to the situation prevailing in human primary CD4^+ ^T cells, up to 50% of Jurkat cells were productively infected with HIV-1 at 5 days post-infection (data not shown).

### HIV-1 rapidly modulates host gene expression

**Figure 2:**
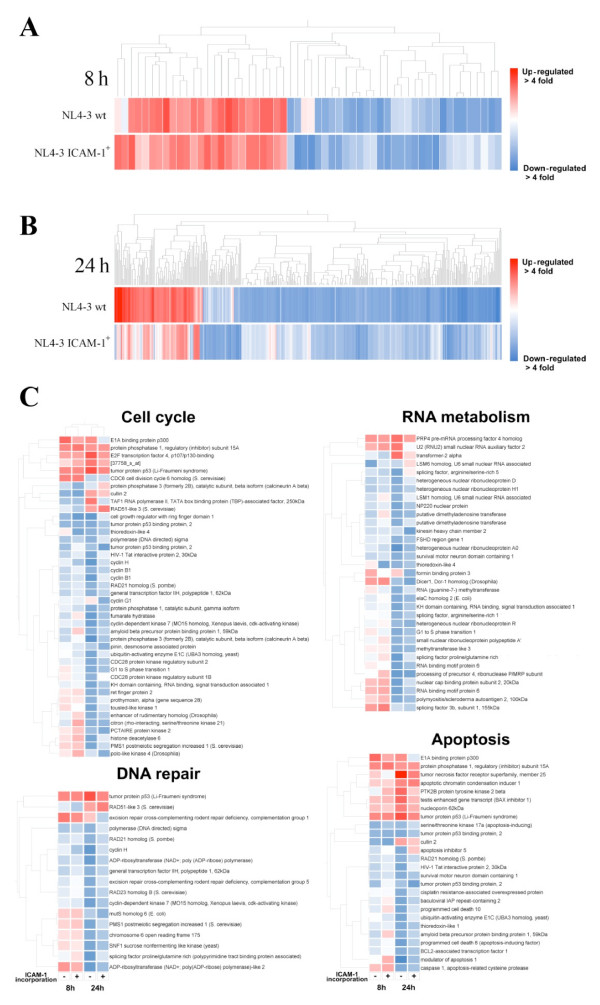
**Hierarchical clustering and gene ontology analysis of microarray data**. (A) A hierarchical clustering of the 56 genes that are modulated (down- or up-regulated) by either NL4-3 wt or NL4-3 ICAM-1+ at 8 h post-infection (as determined by a two-fold threshold) has been defined using the correlation function of GeneSpring 6.0. (B) A hierarchical clustering of the 363 genes that are modulated (down- or up-regulated) by either NL4-3 wt or NL4-3 ICAM-1+ at 24 h post-infection (as determined by a two-fold threshold) has been defined using the correlation function of GeneSpring 6.0. (C) Hierarchical clustering of genes belonging to selected Gene Ontology categories identified by a Gene Ontology overrepresentation analysis as being significantly enriched within the list of genes modulated by HIV-1 is shown.

Having established some characteristics of the studied cell population such as purity and permissiveness to productive viral infection, gene microarray analysis was performed to measure the impact of HIV-1 on host gene expression in CD4^+ ^T lymphocytes. Cells were isolated from five healthy donors and either left uninfected (mock) or infected with isogenic NL4-3 wt or NL4-3 ICAM-1+ for 8 and 24 h (Fig. 1C). Next, RNA was extracted, pooled and processed according to the manufacturer's instructions and then hybridized on HG-U95v2 oligonucleotide arrays (Affymetrix). Gene expression data was obtained with the Affymetrix Microarray Suite software (version 5.0). Analysis of the microarray data revealed that HIV-1 significantly influenced the transcriptomic profile of the cell population enriched in CD4^+ ^T cells in spite of the weak infection rate. Indeed, we determined that, out of the 4,289 genes with a present call in all six arrays, 404 genes were modulated (either up- or down-regulated) at least twofold by either viruses compared to controls. A very limited number of cellular genes were differentially regulated at 8 h post-infection (i.e. 8 genes modulated at least 2 fold by both virus stocks and 56 genes affected by either NL4-3 wt or NL4-3 ICAM-1+) (Additional file 1), whereas the majority of changes were observed at 24 h post-infection (i.e. 22 genes modulated 2 fold or more by both virus preparations and 363 genes by either NL4-3 wt or NL4-3 ICAM-1+) (Additional file 2). Interestingly, 28.5% of the genes modulated either by NL4-3 wt or NL4-3 ICAM-1+ at 8 h post-infection are still affected at the 24 h time point. The large discrepancy between the numbers of genes modulated by both virus preparations and by either of them hinted at large differences between the two viral preparations, suggesting that ICAM-1 had a significant impact on transcriptional profiles of CD4^+ ^T lymphocytes. However, when we compared differences in gene expression between isogenic virions either lacking or bearing host-derived ICAM-1, we found that the majority of the discordant genes were in fact regulated in the same direction (either down- or up-regulated), missing the twofold threshold for either virus as monitored by hierarchical clustering. The correlation is especially good for genes regulated at 8 h post-infection (Fig. 2A). At 24 h post-infection, there is still an excellent correlation between both virus stocks, although the modulation induced by NL4-3 wt is overall greater than for ICAM-1+ viruses (Fig. 2B). This suggests that the faster kinetics of infection with ICAM-1-bearing virions probably result in a faster return of gene expression to normal levels. However, the low number of time points analysed does not allow us to confirm this hypothesis. Nevertheless, the gene expression profiles with ICAM-1-bearing virions are still interesting for two reasons. First, they provide strength to our microarray experimental design as gene expression profiles induced by both viruses are highly similar, thus indicating that genes induced by both viruses are far less susceptible to be false positives. Second, our findings suggest that the HIV-1-mediated gene expression alterations are most likely occurring in uninfected/bystander cells given that a 1.5-fold increase in the number of infected cells is seen at 24 h post-infection with ICAM-1-bearing virions compared to infection with viruses lacking host-derived ICAM-1 while the number of genes affected by NL4-3 wt is higher.

### Multiple biological processes are affected by HIV-1

In order to identify the most dramatically affected biological pathways, we performed statistical Gene Ontology (GO) overrepresentation analysis on the microarray data. This technique identifies biological processes, molecular functions and cellular localization categories that contain a high proportion of modulated genes. This approach is useful for identifying the cellular processes that are the most affected by the tested stimuli and for pointing out biological areas that warrant further studies. A careful analysis revealed that many major biological processes were significantly affected by HIV-1 (*P *< 0.01). Among these, we found that apoptosis, DNA repair, cell cycle and RNA metabolism were the most influenced categories, as determined by the number of modulated genes. A per-category hierarchical cluster of the genes affected by HIV-1 in those categories is depicted in Fig. 2C.

### p53 is transcriptionally up-regulated by HIV-1

**Figure 3:**
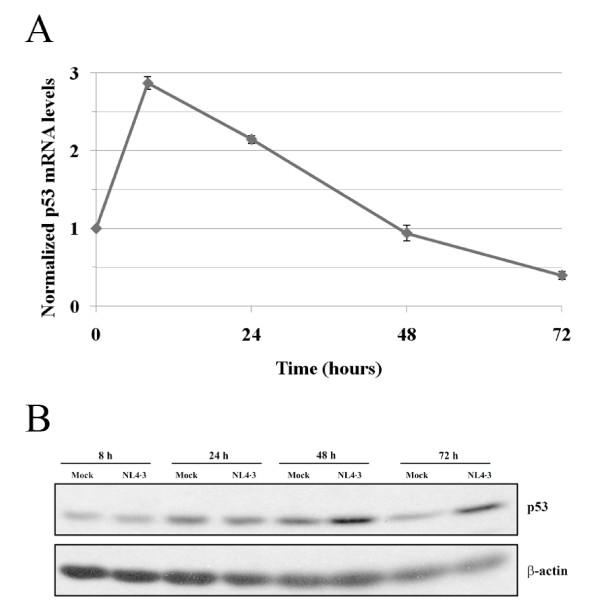
**Quantification of p53 by qRT-PCR and western blot**. (A) The purified cell population highly enriched in CD4^+ ^T lymphocytes was either left uninfected or infected with NL4-3 wt for 8, 24, 48 and 72 h. Total RNA from the five original healthy donors and from six additional donors was isolated. Ribosomal 18S and p53 RNA levels were quantified by qRT-PCR. Data shown is representative of all studied samples (i.e. a total of eleven) normalized on ribosomal 18S. (B) Cells were either left uninfected (mock) or infected with NL4-3 wt for the indicated time periods. Next, p53 and actin protein levels were estimated by western blot analysis using a specific antibody.

A closer analysis of the various genes modulated by HIV-1 revealed that the tumor suppressor gene p53 is present in three out of the four significantly modulated GO categories identified (i.e. apoptosis, DNA damage and cell cycle) and is highly regulated by both viruses at both studied time points. Activation of p53 via phosphorylation has been implicated in HIV-1-induced apoptosis and it has been identified as the dominant apoptosis-inducing factor elicited by the HIV-1 envelope along with the ubiquitous mammalian transcription factor NF-κB [28]. It has been demonstrated that p53 is mostly regulated at the post-transcriptional level by HDM2 but the mechanism(s) by which p53 is regulated at the transcriptional level is still poorly understood [29]. Previous studies linking HIV-1 and p53 refer to a post-transcriptional induction by phosphorylation. Therefore, we found interesting to focus on the unexpected up-regulation of p53 mRNA in our subsequent experiments as its transcriptional regulation by HIV-1 is novel. A quantitative analysis of p53 by qRT-PCR was next performed to confirm microarray data. qRT-PCR data was consistent with the microarray results since p53 was found to be up-regulated by HIV-1 at 8 and 24 h post-infection, returning to basal levels at the 48 h time point (Fig. 3A). Western blot analyses were also performed to examine the impact of HIV-1 on p53 expression at the protein level. This protein was increased by HIV-1 (Figs. 3B and 3C) but at a later time point than expected according to mRNA data. Indeed, the virus-mediated augmentation in p53 protein level was only detected at 24 or 48 h post-infection while p53 mRNA was already enhanced at 8 h post-infection. This pattern of delayed protein production following mRNA up-regulation is well described for p53 as it is regulated by HDM2 at the post-translational level [30].

Other attractive HIV-1-induced candidate genes include GADD34 (also called PP1R15A), which is indirectly involved in p53 regulation via PP1 [31,32], and TNFRSF25, a cell surface receptor that carries a death domain. An increase in mRNA levels similar to microarray data was confirmed by qRT-PCR at 8 and 24 h post-infection for both GADD34 (2.25 fold increase at 8 h and 2.65 fold increase at 24 h) and TNFRSF25 (2.4 fold increase at 8 h and 3.61 fold increase at 24 h) (data not shown). Unfortunately, we could not assess the effect of HIV-1 on these genes at the protein level because the commercial anti-GADD34 and anti-TNFRSF25 antibodies we tested displayed a very weak specificity (data not shown). This severely impaired our ability to define their relevance in the context of HIV-1 infection. We intend to revisit these two candidates as soon as reliable antibodies are commercially available.

### HIV-1-mediated up-regulation of p53 mRNA is associated with secretion of type-I IFN

**Figure 4:**
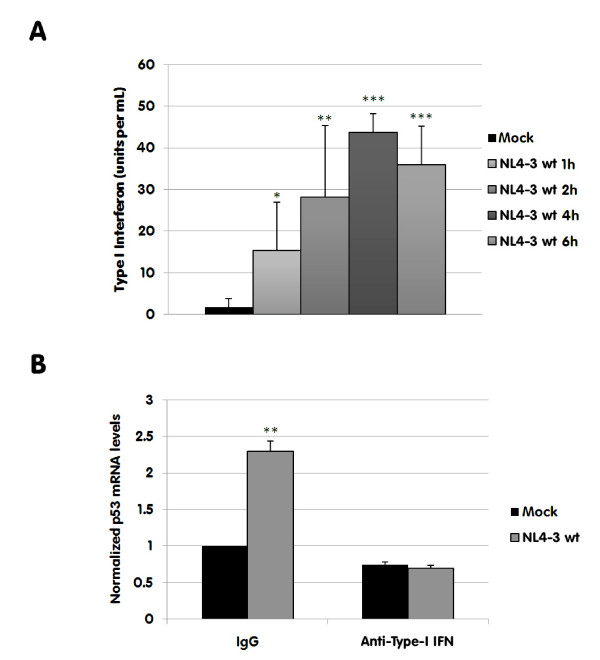
**Virus-induced type-I IFN secretion and blocking experiments with anti-IFN-α and -β antibodies**. (A) Cells were either left uninfected or infected with NL4-3 wt for the indicated times. Thereafter, .HEK-Blue™ IFN-α/β cells were exposed to the collected cell-free supernatants for 24 h and type-I IFN levels were quantified according to manufacturer's instructions. (B) The purified cell population highly enriched in CD4^+ ^T lymphocytes was either left uninfected or infected with NL4-3 wt for 24 h. In some samples, neutralizing antibodies against human IFN-α and IFN-β were added in equal quantities (a final concentration of 1 μg/ml was used). Controls consisted of cells treated with isotype-matched irrelevant antibodies (IgG). Thereafter, total RNA was isolated and p53 mRNA levels were estimated by qRT-PCR. The data shown represents the mean ± standard deviations of triplicate samples and are representative of three independent experiments. Asterisks denote statistically significant differences from the uninfected control cells (*, P < 0.05; **, P < 0.01; ***, P < 0.001).

Next, we investigated the mechanism through which HIV-1 can up-regulate p53 gene expression. The protein is known to be regulated post-transcriptionally by HDM2, which binds to and induces the ubiquitinylation of p53, causing its destruction by the proteasome before it can act as a potent transcription factor and induce apoptosis [29]. Phosphorylation of p53 allows it to escape HDM2 binding leading to its accumulation and activation of its transcription factor capabilities. Although this phenomenon has been previously documented in the context of HIV-1-induced apoptosis, our data suggest that p53 is also regulated at the mRNA level, which represents a distinct and previously uncharacterized process in the context of HIV-1 infection. Takaoka and colleagues have reported that an increase in p53 mRNA can be induced by type-I IFN [33], a process that is associated with antiviral immunity as the up-regulation of p53 mRNA would prepare neighbour cells to undergo apoptosis, preventing the spread of viral infection. It should be noted that the up-regulation of p53 mRNA does not necessarily lead to an immediate up-regulation of the protein, which is still tagged for degradation by HDM2 until it is activated. Instead, the additional mRNA prepares the cells to undergo apoptosis more quickly and efficiently if they are infected. Many viruses were identified in this study as being able to induce IFN-mediated p53 mRNA up-regulation but there was no mention of HIV-1. Thus, we investigated whether the observed increase in p53 mRNA in our experimental cell system was linked to the presence of type-I IFN in our cell cultures. First, we measured the production of such soluble factors in cell-free supernatants following exposure to HIV-1 using ELISA detection kits specific for IFN-α and IFN-β. We found that IFN-α was secreted at very low levels upon HIV-1 infection since the amount of this cytokine was found to be slightly above the detection limit of the ELISA test (i.e. 10 pg/ml) (data not shown). We could not detect the presence of IFN-β when using a commercial ELISA test with a sensitivity of 300 pg/ml (data not shown). Therefore, we used an alternative strategy to measure the seemingly low doses of type-I IFN. To this end we used the HEK-Blue™ IFN-α/β cells that can detect the biologically active form of type-I IFN. As depicted in Fig. 4A, a virus-dependent induction of type-I IFN was seen shortly after exposure of the population highly enriched in CD4^+ ^T cells to HIV-1, which is consistent with the rapid induction of p53 (i.e. 8 h). To corroborate the contribution of IFN-α and IFN-β in the HIV-1-mediated augmentation in p53 gene expression, we used another experimental procedure based on neutralizing antibodies. Data depicted in Fig. 4B indicate that the virus-dependent increase in p53 mRNA is indeed linked with production of type-I IFN (i.e. IFN-α and IFN-β) as the virus-mediated increase in p53 mRNA was completely inhibited in presence of blocking antibodies.

## Discussion

In this study, we used Affymetrix oligonucleotide microarrays as a survey tool to obtain an overview of the transcriptional changes induced by HIV-1 in a population of human primary cells highly enriched in CD4^+ ^T lymphocytes. We also attempted to determine whether the global gene expression pattern could be altered when target cells are interacting with virions bearing host-derived ICAM-1 on their surface as compared to isogenic viruses lacking this host molecule.

Experimental design in microarray studies essentially follows two different strategies. First, a replicate approach where each experimental condition can be biologically repeated multiple times and analyzed on multiple arrays. Second, the pooling approach where RNA from different experiments are combined together and assayed on one array for each condition in an effort to reduce the inherent biological variability. Ideally, the replicate strategy is preferred as it allows statistics to be used to identify significantly modulated genes, controlling and reducing the number of expected false positives. However, according to Pan and co-workers, the statistical power gained from very few replicates (i.e. less than 4) is negligible [34]. For example, it was reported that no less than 4 to 8 replicates per experimental condition are necessary to obtain significant statistical power. Other studies have shown that RNA pooling is a valid alternative to biological replicates [35-37] and that this strategy can provide the same statistical power as the replicates approach [38] at a much reduced cost when appropriate precautions are taken (see Methods section). Therefore, we decided to use the pooling approach to study the HIV-1-mediated changes in gene expression profiles in a cell population highly enriched in CD4^+ ^T cells. The statistical significance and validity of our findings are improved because results with the two virus stocks tested are very similar and can be considered as pseudo-replicates. Indeed, the virus-induced modulation of global gene expression profiles with virions either lacking or bearing host-derived ICAM-1 were found to be comparable.

Characterization of the studied cell subpopulation is crucial in microarray experiments. Ideally, the starting material needs to be as homogenous as possible to avoid a possible contamination with mRNAs from undesirable cells [39,40]. In the present work, we used commercially available CD4^+ ^T cells negative selection kits from Miltenyi Biotec and StemCell Technologies. A negative selection procedure was preferred to avoid any putative antibody-mediated signaling events. Although both manufacturers claim that the purity of the isolated cell population is high (i.e. > 95%), their recommended flow cytometry analysis to assess cell purity only makes use of an antibody against CD4, neglecting the fact that monocytic cells (CD14^+^) can also express a lower level of this cell surface marker. Furthermore, they used frozen-thawed PBMCs, a process that can be deleterious to some CD4-expressing cells such as dendritic cells and their precursors [41]. In our hands, the vast majority of isolated cells were indeed positive for CD4 (i.e. > 96%), but a fraction (i.e. ranging from 5% up to 15% in some rare cases) also expressed CD14, a marker for cells of the monocytic lineage (e.g. monocytes). Although it is generally accepted that peripheral blood monocytes are not productively infected with HIV-1 [42], there is at least one report that monocytes can sustain low levels of HIV-1 replication *in vivo *[43]. This cell type can also indirectly affect gene expression in CD4^+ ^T cells through the production of soluble factors. It should also be specified that plasmacytoid dendritic cells are negative for CD14 but do express CD4. It is unclear whether these cells are present in the studied cell population since they represent a very small proportion of PBMCs (i.e. < 1%) but could have been enriched along with CD4^+ ^T cells as they are not specifically targeted by antibodies of the negative selection kits we used. Interestingly, it has been shown that these cells can rapidly produce very large quantities of type-I IFN following exposure to HIV-1 [44,45].

The virus infection rates seen under our experimental conditions were extremely low in primary human cells compared to Jurkat T lymphoid cells (i.e. at 8 and 24 h post-infection). It is possible that the presence of type-I IFN seen in our cell system could contribute to this low level of virus infection. Given the very low percentage of cells productively infected with HIV-1 and considering that there were minor differences in gene expression profiles following infection with NL4-3 wt and NL4-3 ICAM-1+, it would thus be unlikely that the observed modulation of host-cell gene expression is occurring exclusively in cells productively infected with HIV-1. Therefore, it can be proposed that the vast majority of alterations of the gene expression profiles seen in this study are most likely taking place in uninfected/bystander cells. One way to elucidate whether the differential gene expression pattern is seen in HIV-1-infected and/or uninfected/bystander cells would be to infect target cells with replication competent HIV-1 that would contain all viral genes but would code also for a distinctive cell surface molecule. This tool would allow isolation of cells productively infected with HIV-1 from bulk populations of cells and a large scale monitoring of host cell gene expression in both virus-infected and uninfected/bystander cells.

Analysis of modulated genes by a Gene Ontology-based approach revealed that several major pathways were affected by HIV-1 including apoptosis, RNA metabolism, DNA repair and cell cycle. Interestingly, Corbeil and colleagues concluded that HIV-1 affects expression of genes involved in DNA repair and apoptosis [46]. They suggested that HIV-1 induces a DNA repair response following its integration that ultimately leads to p53 activation and caspase-dependent apoptosis. It has been established that activation of p53 relies on its phosphorylation [47]. This activation results in induction of the pro-apoptotic factor Bax and depolarization of mitochondrial membranes, followed by caspase activation [48]. However, they did not observe regulation of p53 at the mRNA level even if they reported an increase of p53 at the protein level following its phosphorylation. The fact that they used an established cell line (i.e. CEM-GFP) instead of primary human cells could account for the discrepant results. Although some established cell lines display a higher susceptibility to productive HIV-1 infection than primary human cells, the former can harbour multiples deficiencies in critical cellular pathways such as apoptosis, DNA repair or cell cycle regulation. Thus, it is difficult to compare our results with previous microarray studies involving HIV-1. Even for studies using primary cells, small differences in experimental setup or the source of cells (i.e. peripheral CD4^+ ^T lymphocytes versus CD4^+ ^T cells isolated from lymphoid organ) can account for discrepancies observed when such comparisons are made. Direct comparison with large-scale proteomic studies such as those published by Ringrose and Chan [49,50] are even more problematic, as multiple layers of post-traductional and post-translational regulation likely come into play after mRNA modulation.

We focused our efforts on characterizing the up-regulation of p53 at the mRNA level, which is an uncommon phenomenon as the protein is highly regulated at the post-transcriptional level. Moreover, its transcriptional regulation was previously uncharacterized in the context of HIV-1. Our interest for p53 was prompted by the relatively high number of genes we found to be regulated by HIV-1 in the microarray experiment that interact with p53 either directly or indirectly, such as HIV-1 Tat interacting protein (HTATIP2), p300, GADD34 and TP53BP2. p53 is also known to interact directly or indirectly with several HIV-1 proteins such as Tat [51], Nef [52], reverse transcriptase [28] and Vpr [53]. Some of these interactions can inhibit the function of p53 as a transcription factor, leading to a reduced sensitivity to apoptosis in infected cells, which can be considered as beneficial for the virus survival. On the other hand, the precise effect of p53 with respect to the viral promoter region is still unclear. Some reports claim that p53 is essential for efficient viral transcription [54,55], while others suggest that p53 can negatively influence transcription from the viral promoter by inhibiting the transduction activity of Tat [56,57].

The p53-related gene GADD34 was identified as another interesting candidate for future studies as GADD34 is a PP1 subunit that impairs p53 dephosphorylation [32]. PP1 is one of the phosphatases responsible for dephosphorylating p53 [31], maintaining a delicate balance between survival and apoptosis. Therefore, an up-regulation of GADD34 might facilitate phosphorylation of p53, which will in turn promote apoptosis in CD4^+ ^T cells. Another candidate of potential interest is TP53BP2, a p53-binding gene that codes for two distinct proteins through differential splicing, namely 53BP2S and 53BP2L (also known as ASPP2) [58]. The biological significance of this differential splicing is not yet well characterized. Both isoforms can bind p53 [59], Bcl-2 [60] and the p65 subunit of NF-κB [61]. Interestingly, it appears that TP53BP2 can also bind PP1 and interferes with p53 dephosphorylation [62]. However, the late discovery of the second isoform led to confusion and controversy about the biological role and molecular function of TP53BP2. It has been proposed that binding of TP53BP2 to p53 inhibits its potency as a pro-apoptotic transcription factor [63], while others have shown that overexpression of TP53BP2 results in apoptosis [64]. Comprehensive studies on those two promising candidates were not carried out because commercial antibodies of good quality are not available. We plan to evaluate the role played by GADD34 and both isoforms of TP53BP2 in regard to HIV-1 and its relation with p53 in the near future.

An elegant study has documented a mechanism involved in transcriptional regulation of p53 that is mediated by type-I IFN in response to viruses [33]. Moreover, a recent study has shown that p53 itself can positively regulate IFN-mediated signalling events and production from infected cells [65], adding further evidence of the importance of p53 in the antiviral response. Although the type-I IFN-mediated p53 mRNA induction has been characterized for many viruses, there is still no information with respect to the importance of this process in the pathobiology of HIV-1. Thus, we decided to assess the involvement of type-I IFN in the HIV-1-mediated up-regulation of p53 mRNA. Results showed induction of type-I IFN by HIV-1 in our cell culture system and a complete inhibition of the HIV-1-mediated increase in p53 gene expression in presence of a combination of blocking antibodies specific for type-I IFN (i.e. IFN-α and IFN-β). This clearly establishes a novel and direct link between p53, HIV-1 and type-I IFN. Such a mechanism constitutes an essential part of the antiviral immune response by increasing the intracellular pool of p53 mRNA in response to virus infection. This process is aimed at preventing the spread of infection by allowing a more rapid induction of apoptosis. It is important to note that an increase in p53 mRNA has no immediate effect on the total p53 protein levels as the latter is continuously degraded by the proteasome back to steady state levels. Indeed, an additional signal such as DNA damage is required to activate p53 by phosphorylation, causing its escape from HDM2-induced degradation and translocation to the nucleus where it can induce transcription of pro-apoptotic genes. This might help to explain the delay seen between the HIV-1-mediated induction of p53 expression at the mRNA and protein levels. Indeed, a 3 to 5-fold increase in p53 mRNA was detected as early as 8 h following HIV-1 infection while an induction of p53 at the protein level could be detected only after 24 to 48 h following exposure to HIV-1, depending on the donor. This suggests that post-translational control mechanisms such as HDM2-mediated ubiquitinylation and proteasome-dependent degradation of p53 at first counteract the increase at the mRNA level. It can be proposed that the type-I IFN-mediated induction of p53 mRNA serves to avoid an uncontrolled up-regulation of this protein in every single cell exposed to type-I IFN as this would lead to massive and indiscriminate induction of apoptosis. Instead, it can be postulated that an increase in p53 mRNA might prepare cells to undergo apoptosis rapidly would the presence of an incoming menace (such as HIV-1 or other retroviruses that can integrate within the host genome) materialize. Indeed, following exposure to type-I IFN, a faster apoptosis response in cells exposed to DNA damage would be triggered as the larger p53 mRNA pool would rapidly lead to more p53 protein. This would induce a stronger activation of pro-apoptotic genes once p53 escape the control of HDM2-mediated ubiquitinylation and proteasome-dependent degradation following its phosphorylation.

It has been reported that CD4^+^/CD14^+ ^cells (e.g. monocytes) can produce type-I IFN in response to HIV-1 [12]. Also, it has been shown that HIV-1 can cause massive type-I IFN production from plasmacytoid dendritic cells (PDCs) [66]. It should be stated that this dendritic cell subtype is short-lived under *in vitro *conditions without the appropriate cytokines cocktail and constitutes a very low percentage of the total PBMCs (i.e. < 1%). However, in a study establishing a link between TRAIL induction and HIV-1, Audigé and colleagues identified that a minimal contamination of their CD4 population isolated from tonsils with PDCs (less than 0.5%) was sufficient to produce enough type-I IFN to induce a TRAIL-dependent apoptotic process [16]. It is highly probable that a similar contamination is at least partly responsible for the presence of type-I IFN in our experimental system. It can thus be proposed that the observed type-I IFN secretion in the present study is due to contaminating monocytes and/or PDCs. *In vivo*, other cell types can produce type-I IFN in response to viruses and could contribute to the observed phenomenon.

**Figure 5:**
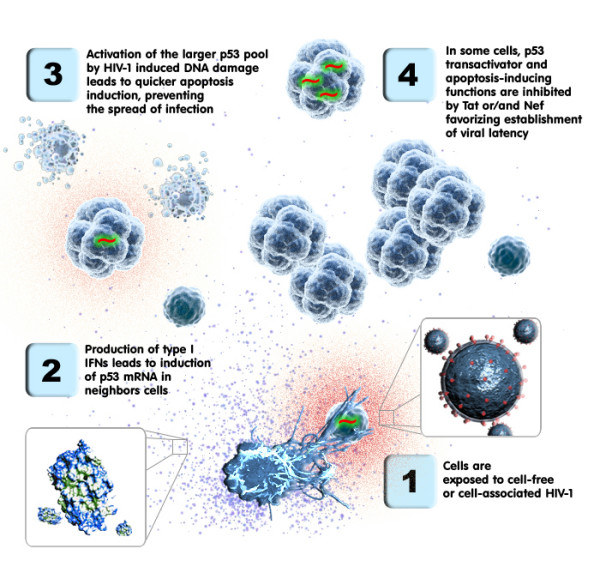
**Proposed working model**. This diagram depicts the possible sequence of events that is initiated following exposure of a cell population highly enriched in CD4^+ ^T cells to HIV-1. The fourth step is hypothetical and is derived from data collected in the literature.

Based on the data we collected and previously published studies, we propose the following hypothetical model (Fig. 5). Exposure of human primary cells highly enriched in CD4^+ ^T cells to HIV-1 leads to a rapid production of soluble factors such as type-I IFN and possibly other soluble factors. This will result in engagement of various signalling cascades and induction of several genes, including p53, GADD34 and TNFRSF25 along with other genes involved in apoptosis, DNA damage, RNA metabolism and cell cycle. The increase in p53 mRNA will not immediately affect p53 protein levels. Eventually, following its activation by signals such as HIV-1-induced DNA damage, p53 will escape HDM2 control, and quickly accumulate due to the additional mRNA induced by type-I IFN. Finally, p53 will concentrate within the nucleus and promote transcription of pro-apoptotic genes such as Bax, ultimately leading to induction of apoptosis. As discussed above, viral proteins that can bind p53 might inhibit its pro-apoptotic activity, allowing more cells to become either productively or latently infected with HIV-1 [52]. It can be postulated that the type-I IFN-mediated increase in p53 mRNA could have evolved to overcome the capability of some viruses to inhibit p53.

The findings presented in this paper have implications in the context of the recently reported HIV-1-induced bacterial translocation. It has been proposed that HIV-1 permeabilizes the gut allowing for bacterial products such as lipopolysaccharide to circulate in the peripheral blood resulting in secretion of type-I IFN [67]. This phenomenon could lead to a sustained increase in p53 mRNA levels and therefore to a higher susceptibility of CD4^+ ^T cells to pro-apoptotic signals such as HIV-1-induced DNA damage.

In conclusion, we confirm that microarrays represent a useful tool for elucidating the molecular details of the complex interaction between HIV-1, its target cells and uninfected/bystander cells. We demonstrate that even small scale gene expression profiling can lead to a better comprehension of host-defence strategies, which is essential for the design of a new generation of therapeutic agents.

## Competing interests

The authors declare that they have no competing interests.

## Authors' contributions

MI designed and carried out all the experiments, and drafted the original manuscript. MO participated in harvesting cells for initial experiments and was involved in experimental design, result analysis and discussions throughout the study. MJT supervised and coordinated the study and finalized the manuscript. All authors read and approved the manuscript.

## Supplementary Material

Additional file 1 Table S1. Genes modulated at 8 h post-infection. Click here for file

Additional file 2 Table S2. Genes modulated at 24 h post-infection. Click here for file
