# Supplementary material for: Microarray study reveals that HIV-1 induces rapid type-I interferon-dependent p53 mRNA up-regulation in human primary CD4+ T cells
Source: Retrovirology. 2009 Jan 15;6:5. doi: 10.1186/1742-4690-6-5 (PMC2637825; doi:10.1186/1742-4690-6-5)
Supplement: Additional file 1 — Table S1. Genes modulated at 8 h post-infection. [file 1742-4690-6-5-S1.doc]

**Table S1: Genes modulated at 8 h post-infection.**

| **Affymetrix ID** | **Gene name** | **Genbank** | **NL4-3 wt** | **NL4-3 ICAM-1+** |
| --- | --- | --- | --- | --- |
| **Genes modulated by both viruses – “High confidence”** | | | | |
| 40332_at | OGFR | AF109134 | 2.44 | 2.517 |
| 40495_at | CBCIP2 | AA306076 | 2.824 | 2.007 |
| 35626_at | SGSH | U30894 | 2.417 | 2.288 |
| 36598_s_at | INPPL1 | L36818 | 2.363 | 2.055 |
| 37028_at | PPP1R15A | U83981 | 2.005 | 2.171 |
| 35247_at | SNAPC5 | AI557062 | 0.383 | 0.443 |
| 37894_at | CUL2 | U83410 | 0.492 | 0.419 |
| 40852_at | PCTAIRE2BP | AB025254 | 0.482 | 0.485 |
| **Genes modulated by either viruses – ordered by confidence** | | | | |
| 1974_s_at | TP53 | X02469 | 2.005 | 1.892 |
| 41784_at | DKFZp564B0769 | AL080186 | 2.043 | 1.802 |
| 1131_at | MAP2K2 | L11285 | 1.816 | 2.176 |
| 32822_at | SLC25A4 | J02966 | 0.562 | 0.462 |
| 38139_at | FPGT | AF017445 | 1.733 | 2.123 |
| 1878_g_at | ERCC1 | M13194 | 2.151 | 1.747 |
| 39444_at | SF3B1 | AF054284 | 1.666 | 2.016 |
| 37737_at | PCMT1 | D25547 | 1.955 | 2.54 |
| 38823_s_at | STK17A | AI961743 | 0.495 | 0.606 |
| 187_at | MAP4K2 | U07349 | 2.078 | 1.681 |
| 40027_at | ATP5S | W52999 | 1.65 | 2.067 |
| 40479_at | FYN | Z97989 | 0.609 | 0.475 |
| 38325_at | MINPP1 | AL050356 | 0.571 | 0.416 |
| 41308_at | CTBP1 | U37408 | 2.325 | 1.671 |
| 193_at | TAF9 | U21858 | 0.487 | 0.648 |
| 41565_at | A2LP | AF034373 | 2.096 | 1.559 |
| 1764_s_at | MAZ | D85131 | 2.365 | 1.598 |
| 36514_at | CGR19 | U66469 | 0.555 | 0.339 |
| 40088_at | NRIP1 | X84373 | 0.583 | 0.365 |
| 1146_at |  |  | 1.558 | 2.358 |
| 36927_at | C1orf29 | AB000115 | 0.647 | 0.43 |
| 40702_at | IFNG | X13274 | 0.426 | 0.656 |
| 39576_at | PDE6B | S41458 | 0.636 | 0.395 |
| 37485_at | SLC27A2 | D88308 | 0.441 | 0.674 |
| 33071_at |  | Z98744 | 0.341 | 0.597 |
| 37425_g_at | C6orf18; HCR | AB029343 | 2.205 | 1.445 |
| AFFX-HUMISGF3A | STAT1 | M97935 | 0.45 | 0.691 |
| 33930_at | RA410 | AB020724 | 0.458 | 0.704 |
| 551_at | EP300 | U01877 | 2.437 | 1.469 |
| 33934_at | SUSP1 | AB018340 | 0.496 | 0.747 |
| 41459_at | TPP2 | M73047 | 2.766 | 1.513 |
| 37453_at | CLPX | AJ006267 | 0.466 | 0.735 |
| 33399_at |  | AA142942 | 0.759 | 0.498 |
| 38277_at | PPP3CB | M29550 | 0.766 | 0.479 |
| 32065_at | CREM | S68134 | 0.483 | 0.785 |
| 41823_at | STAU | AJ132258 | 0.481 | 0.789 |
| 39324_at |  | AL050078 | 0.806 | 0.484 |
| 1560_g_at | PAK2 | U24153 | 0.398 | 0.771 |
| 34491_at | OASL | AJ225089 | 2.019 | 1.217 |
| 35991_at | LSM6 | AA917945 | 0.414 | 0.798 |
| 33352_at | H2BFQ | X57985 | 0.421 | 0.833 |
| 41212_r_at | WBSCR1 | D26068 | 0.463 | 0.859 |
| 36334_at | LY9 | L42621 | 2.001 | 1.12 |
| 41745_at | IFITM3 | X57352 | 0.448 | 0.939 |
| 36090_at | TBL2 | AL080162 | 1.045 | 2.213 |
| 35245_at | F5 | M16967 | 1.022 | 0.349 |
| 33631_at | DIM1 | AF023612 | 1.031 | 0.402 |
| 36998_s_at | SCA2 | Y08262 | 0.924 | 2.072 |
