# Supplementary material for: Microarray study reveals that HIV-1 induces rapid type-I interferon-dependent p53 mRNA up-regulation in human primary CD4+ T cells
Source: Retrovirology. 2009 Jan 15;6:5. doi: 10.1186/1742-4690-6-5 (PMC2637825; doi:10.1186/1742-4690-6-5)
Supplement: Additional file 2 — Table S2. Genes modulated at 24 h post-infection. [file 1742-4690-6-5-S2.doc]

**Table S2: Genes modulated at 24 h post-infection.**

| **Gene Name** | **Common** | **Genbank** | **NL4-3 wt** | NL4-3 ICAM-1+ |
| --- | --- | --- | --- | --- |
| **Genes modulated by both viruses – “High confidence”** | | | | |
| 1331_s_at | TNFRSF25 | U83598 | 4.976 | 2.057 |
| 31829_r_at | TGOLN2 | AF027515 | 2.661 | 2.012 |
| 33398_at | ACINUS | AB014570 | 2.13 | 2.021 |
| 949_s_at | PSMC6 | D78275 | 0.336 | 0.344 |
| 706_at |  |  | 0.327 | 0.37 |
| 40563_at | NUP50 | AL050007 | 0.369 | 0.357 |
| 38380_at | POP4 | Y18863 | 0.36 | 0.399 |
| 37751_at | KIAA0255 | D87444 | 0.355 | 0.405 |
| 35614_at | TCFL5 | AB012124 | 0.326 | 0.472 |
| 37842_at | HIC | AF054589 | 0.314 | 0.489 |
| 37177_at | CD58 | Y00636 | 0.313 | 0.491 |
| 32827_at | RRAS2 | AI365215 | 0.396 | 0.417 |
| 39086_g_at | SSBP1 | AA768912 | 0.361 | 0.452 |
| 38355_at | DBY | AF000984 | 0.348 | 0.468 |
| 869_at | GTF2A2 | U14193 | 0.428 | 0.407 |
| 39091_at | JWA | AF070523 | 0.455 | 0.39 |
| 32697_at | IMPA1 | AF042729 | 0.434 | 0.417 |
| 38160_at | LY75 | AF011333 | 0.385 | 0.482 |
| 36932_at | GTF3C2 | D13636 | 0.406 | 0.496 |
| 35811_at | RNF13 | AF037204 | 0.47 | 0.453 |
| 33514_at | CAMK4 | D30742 | 0.48 | 0.5 |
| 35824_at | ZNF238 | AJ223321 | 0.492 | 0.499 |
| **Genes modulated by either viruses – ordered by confidence** | | | | |
| 32564_at | SEC61B | AA083129 | 0.509 | 0.49 |
| 37358_at | UBE2E1 | AI039880 | 0.484 | 0.507 |
| 41834_g_at | JTB | AB016492 | 0.483 | 0.512 |
| 32245_at | METTL3 | AF014837 | 0.493 | 0.524 |
| 36023_at | PRH1 | AI864120 | 0.495 | 0.53 |
| 41569_at | DNAJC9 | AI680675 | 0.518 | 0.482 |
| 31895_at | BACH1 | AB002803 | 0.482 | 0.523 |
| 39128_r_at | PPP2R4 | X73478 | 2.167 | 1.972 |
| 33351_at | GC20 | AF064607 | 0.482 | 0.529 |
| 40509_at | ETFA | J04058 | 0.544 | 0.495 |
| 34785_at | KIAA1025 | AB028948 | 0.487 | 0.538 |
| 39367_at | ELAC2 | AA522537 | 0.469 | 0.525 |
| 41557_at | KIAA0052 | D29641 | 0.486 | 0.542 |
| 38072_at | DJ465N24.2.1 | AL031432 | 0.488 | 0.547 |
| 41638_at | KIAA0073 | D38552 | 0.469 | 0.53 |
| 1857_at | MADH7 | AF010193 | 0.516 | 0.454 |
| 33438_at | WBP2 | AL049981 | 2.09 | 1.833 |
| 41438_at | OSBPL8 | AL049923 | 0.506 | 0.433 |
| 40063_at | NDP52 | U22897 | 0.463 | 0.54 |
| 39000_at | NMT1 | AF043324 | 2.031 | 1.746 |
| 39219_at | CEBPG | U20240 | 0.542 | 0.456 |
| 34355_at | MECP2 | AJ132917 | 0.418 | 0.51 |
| 34994_at | N143 | AJ002572 | 2.184 | 1.818 |
| 33932_at | GSPT1 | X17644 | 0.48 | 0.573 |
| 36649_at | PMSCL2 | X66113 | 0.462 | 0.557 |
| 38050_at | BTF | D79986 | 0.499 | 0.591 |
| 40852_at | PCTAIRE2BP | AB025254 | 0.58 | 0.484 |
| 34394_at | ADNP | AB018327 | 0.496 | 0.591 |
| 39792_at | HNRPR | AF000364 | 0.586 | 0.489 |
| 41219_at | KIAA0570 | AL050376 | 0.455 | 0.558 |
| 37609_at | NUBP1 | U01833 | 0.479 | 0.58 |
| 38712_at | C1orf9 | AL035291 | 0.549 | 0.443 |
| 37725_at | PPP1CC | X74008 | 0.486 | 0.588 |
| 574_s_at | CASP1 | M87507 | 0.477 | 0.58 |
| 37793_r_at | RAD51L3 | AF034956 | 1.695 | 2.053 |
| 40931_at | CGI-100 | AL080084 | 0.415 | 0.528 |
| 41669_at | KIAA0191 | D83776 | 0.452 | 0.565 |
| 38443_at | MGC14433 | U79291 | 0.494 | 0.603 |
| 39793_at | GBAS | AF029786 | 0.469 | 0.581 |
| 34060_g_at | PVT1 | AA586695 | 2.224 | 1.77 |
| 33895_at | SH3YL1 | AL050373 | 0.527 | 0.402 |
| 324_f_at |  |  | 0.457 | 0.577 |
| 32624_at | TULIP1 | AL050050 | 0.465 | 0.585 |
| 32857_at | SOS2 | L13858 | 0.465 | 0.586 |
| 379_at | APACD | AB006679 | 0.37 | 0.501 |
| 34371_at | PPP4R1 | U79267 | 0.612 | 0.493 |
| 32214_at | TXNL | AF003938 | 0.48 | 0.604 |
| 39790_at | ATP2A2 | M23115 | 0.608 | 0.483 |
| 38669_at | SLK | D86959 | 0.441 | 0.573 |
| 948_s_at | PPID | D63861 | 0.553 | 0.416 |
| 945_at | PSMD7 | D50063 | 0.498 | 0.625 |
| 32536_at | EBP | Z37986 | 0.464 | 0.596 |
| 36048_at | ZFP318 | AB015342 | 0.506 | 0.363 |
| 39797_at | KIAA0349 | AB002347 | 0.455 | 0.589 |
| 40071_at | CYP1B1 | U03688 | 0.45 | 0.585 |
| 31823_at | CUTL1 | M74099 | 0.488 | 0.619 |
| 891_at | YY1 | M77698 | 0.486 | 0.618 |
| 32830_g_at | TIMM17A | X97544 | 0.479 | 0.614 |
| 31853_at | EED | AF080227 | 0.495 | 0.628 |
| 35239_at | EMD | X86810 | 1.605 | 2.048 |
| 35279_at | TAX1BP1 | U33821 | 0.426 | 0.57 |
| 39162_at |  | AA156987 | 0.497 | 0.631 |
| 35827_at | KIAA0905 | AB020712 | 0.452 | 0.593 |
| 40189_at | SET | M93651 | 0.489 | 0.627 |
| 41573_at | SP3 | X68560 | 0.421 | 0.569 |
| 39039_s_at | UBE2J1 | AI557497 | 0.5 | 0.637 |
| 35744_at | KIAA0141 | D50931 | 0.473 | 0.618 |
| 41718_g_at | FADS1 | AC004770 | 2.126 | 1.622 |
| 32113_at | AIM1 | U83115 | 0.63 | 0.486 |
| 40690_at | CKS2 | X54942 | 0.481 | 0.626 |
| 35734_at |  | AI935551 | 0.459 | 0.608 |
| 36162_at | BSG | X64364 | 2.158 | 1.634 |
| 36163_at | DLD | L13761 | 0.424 | 0.579 |
| 36687_at | COX7B | N50520 | 0.398 | 0.558 |
| 34756_g_at | ADPRTL2 | AJ236876 | 0.385 | 0.547 |
| 39338_at | S100A10 | AI201310 | 0.422 | 0.58 |
| 36202_at | PKIA | S76965 | 0.537 | 0.373 |
| 39856_at | RPL36AL | AI708983 | 0.48 | 0.63 |
| 38306_at | BIG1 | AA477576 | 0.437 | 0.594 |
| 40786_at | PPP2R5C | U37352 | 0.437 | 0.594 |
| 37565_at | MMD | X85750 | 0.374 | 0.54 |
| 35643_at | NUCB2 | X76732 | 0.466 | 0.62 |
| 32171_at | EIF5 | AL080102 | 0.496 | 0.645 |
| 31526_f_at | USP6 | X63547 | 0.478 | 0.631 |
| 31584_at | TPT1 | X16064 | 0.48 | 0.633 |
| 41855_at | HAT1 | AF030424 | 0.414 | 0.578 |
| 37520_at | HSA6591 | AJ006591 | 0.446 | 0.606 |
| 40108_at | BZW1 | D13630 | 0.441 | 0.602 |
| 32674_at | NP220 | D83032 | 0.456 | 0.616 |
| 37962_r_at | STXBP3 | D63506 | 0.648 | 0.492 |
| 40870_g_at | RBM6 | AF069517 | 0.418 | 0.588 |
| 38354_at | CEBPB | X52560 | 0.485 | 0.645 |
| 39073_at | NME1 | AL038662 | 0.473 | 0.637 |
| 37737_at | PCMT1 | D25547 | 0.645 | 0.481 |
| 39873_at | PCTK2 | X66360 | 0.607 | 0.434 |
| 35519_at | FLJ20619 | AL049431 | 2.086 | 1.551 |
| 34387_at | KIAA0205 | D86960 | 0.404 | 0.583 |
| 969_s_at | USP9X | X98296 | 0.475 | 0.644 |
| 36579_at | UBE4A | D50916 | 0.474 | 0.646 |
| 38837_at | DJ971N18.2 | W26226 | 0.592 | 0.408 |
| 40092_at | BAZ2A | AB002312 | 2.256 | 1.608 |
| 36987_at | LMNB2 | M94362 | 2.025 | 1.504 |
| 37031_at | C9orf10 | D80005 | 0.459 | 0.639 |
| 34677_f_at | LOC220594 | AJ012755 | 0.456 | 0.637 |
| 40859_at | FLJ11806 | AI561196 | 0.463 | 0.643 |
| 40571_at | MYO5A | U90942 | 0.338 | 0.537 |
| 1974_s_at | TP53 | X02469 | 3.093 | 1.907 |
| 39346_at | KHDRBS1 | M88108 | 0.454 | 0.637 |
| 38424_at | MBC2 | AB018290 | 0.461 | 0.643 |
| 33656_at | RPL37 | D23661 | 0.437 | 0.628 |
| 32546_at | FH | U59309 | 0.377 | 0.578 |
| 2010_at | SKP1A | U33760 | 0.422 | 0.616 |
| 39140_at | DDXx | AL079292 | 0.356 | 0.56 |
| 35318_at | KIAA0475 | AB007944 | 0.442 | 0.633 |
| 39686_g_at | E46L | AL050282 | 0.391 | 0.591 |
| 37342_s_at | IVD | AF070531 | 2.068 | 1.502 |
| 34326_at | COPB | X82103 | 0.441 | 0.635 |
| 33317_at | CDK7 | L20320 | 0.402 | 0.604 |
| 34224_at | FADS3 | AC004770 | 1.516 | 2.141 |
| 37334_at | HNRPA0 | U23803 | 0.311 | 0.534 |
| 35327_at | EIF3S3 | U54559 | 0.469 | 0.667 |
| 41784_at | DKFZp564B0769 | AL080186 | 0.497 | 0.689 |
| 35349_at | COPS3 | AF031647 | 0.435 | 0.642 |
| 40869_at | RBM6 | AF069517 | 0.429 | 0.641 |
| 39392_at | GNPAT | AJ002190 | 0.495 | 0.692 |
| 35245_at | F5 | M16967 | 0.659 | 0.449 |
| 36542_at | SLC9A6 | AF030409 | 0.482 | 0.686 |
| 38764_at | DICER1 | AF007142 | 0.628 | 0.405 |
| 36201_at | GLO1 | D13315 | 0.489 | 0.693 |
| 193_at | TAF9 | U21858 | 0.431 | 0.65 |
| 34445_at | KIAA0471 | AB007940 | 0.489 | 0.695 |
| 35769_at | GPR56 | AJ011001 | 1.446 | 2.076 |
| 36578_at | BIRC2 | U37547 | 0.459 | 0.675 |
| 37935_at | PRPF4 | AF016369 | 2.457 | 1.569 |
| 2003_s_at | MSH6 | U28946 | 0.694 | 0.482 |
| 40610_at | ZFR | AI743507 | 0.7 | 0.489 |
| 39028_at | KPNB3 | Y08890 | 0.452 | 0.674 |
| 37845_at | HEM1 | M58285 | 0.483 | 0.699 |
| 812_at | PPP1R2 | U68111 | 0.71 | 0.498 |
| 32706_at | HIRA | X89887 | 0.445 | 0.672 |
| 33457_at | RAP140 | AB029028 | 0.36 | 0.606 |
| 32672_at |  | AL049387 | 0.367 | 0.616 |
| 36122_at | PSMA6 | X59417 | 0.449 | 0.68 |
| 38415_at | PTP4A2 | U14603 | 0.465 | 0.693 |
| 41574_at | PNN | Y09703 | 0.712 | 0.492 |
| 36088_at | DSCR2 | AJ006291 | 0.659 | 0.415 |
| 1860_at | TP53BP2 | U58334 | 0.621 | 0.366 |
| 37692_at | DBI | AI557240 | 0.388 | 0.641 |
| 37389_at | SMAP | AI346580 | 0.446 | 0.685 |
| 40843_at | ICAP-1A | AF012023 | 0.701 | 0.468 |
| 40332_at | OGFR | AF109134 | 2.646 | 1.576 |
| 36013_at | C4orf1 | AF006621 | 0.451 | 0.69 |
| 1703_g_at | E2F4 | S75174 | 2.913 | 1.641 |
| 36629_at | DSIPI | AI635895 | 0.464 | 0.703 |
| 1707_g_at | ARAF1 | U01337 | 2.816 | 1.607 |
| 33930_at | RA410 | AB020724 | 0.31 | 0.593 |
| 35760_at | ATP5H | AF087135 | 0.378 | 0.65 |
| 34767_at | MOAP1 | AI670788 | 0.251 | 0.545 |
| 41757_at | VAPB | W25933 | 0.492 | 0.733 |
| 41214_at | RPS4Y | M58459 | 0.397 | 0.669 |
| 38971_r_at | TNIP1 | AJ011896 | 2.726 | 1.546 |
| 37475_at | DKFZP434J046 | AC004144 | 2.072 | 1.371 |
| 37403_at | ANXA1 | X05908 | 0.477 | 0.726 |
| 40903_at | ATP6IP2 | AL049929 | 0.302 | 0.596 |
| 674_g_at | MTHFD1 | J04031 | 0.417 | 0.69 |
| 38917_at | TRD@ | X73617 | 0.4 | 0.678 |
| 33433_at | DKFZP564F0522 | AL049943 | 0.364 | 0.652 |
| 39780_at | PPP3CB | M29551 | 0.312 | 0.611 |
| 31573_at | RPS25 | M64716 | 0.458 | 0.72 |
| 32212_at | PDCD8 | AL049703 | 0.398 | 0.679 |
| 39169_at | SEC61G | AF054184 | 0.345 | 0.64 |
| 853_at | NFE2L2 | S74017 | 0.722 | 0.456 |
| 37758_s_at | TFDP1 | W28479 | 2.272 | 1.405 |
| 38997_at | SLC25A1 | X96924 | 2.604 | 1.483 |
| 41146_at | ADPRT | J03473 | 0.383 | 0.674 |
| 32841_at | ZNF9 | U19765 | 0.468 | 0.732 |
| 39740_g_at | NACA | AF054187 | 0.418 | 0.699 |
| 40957_at | JJAZ1 | D63881 | 0.449 | 0.72 |
| 39274_at | NUP62 | X58521 | 2.163 | 1.372 |
| 35645_at |  | AL050148 | 0.444 | 0.721 |
| 34491_at | OASL | AJ225089 | 0.743 | 0.477 |
| 39767_at | CCT8 | D13627 | 0.459 | 0.733 |
| 38659_at | SHOC2 | AB020669 | 0.449 | 0.729 |
| 36858_at | RRS1 | D25218 | 0.704 | 0.411 |
| 39443_s_at | COX5B | M19961 | 0.472 | 0.745 |
| 37685_at | PICALM | U45976 | 0.41 | 0.705 |
| 40258_at | CSNK2A1 | M55265 | 0.762 | 0.499 |
| 34796_at | TRAM | X63679 | 0.305 | 0.632 |
| 41790_at | ALDH5A1 | AL031230 | 0.428 | 0.721 |
| 39519_at | KIAA0692 | AB014592 | 0.38 | 0.692 |
| 36280_at | GZMK | U26174 | 0.428 | 0.725 |
| 32775_r_at | PLSCR1 | AB006746 | 0.732 | 0.438 |
| 37034_at | ANP32A | U73477 | 0.292 | 0.629 |
| 39785_at | KIAA0092 | D42054 | 0.409 | 0.715 |
| 2075_s_at | MAP2K3 | L36719 | 2.247 | 1.353 |
| 38058_at | DPT | Z22865 | 2.214 | 1.345 |
| 32026_s_at | PDZ-GEF1 | AB002311 | 0.468 | 0.757 |
| 525_g_at | PMS1 | U13695 | 0.453 | 0.749 |
| 40486_g_at | TRIM44 | AA176780 | 0.46 | 0.758 |
| 34886_at | RDX | L02320 | 0.763 | 0.465 |
| 37391_at | CTSL | X12451 | 0.453 | 0.757 |
| 33936_at | GALC | D86181 | 0.429 | 0.743 |
| 40826_at | MARK3 | M80359 | 0.442 | 0.752 |
| 40100_at | ARHGEF2 | U72206 | 2.52 | 1.379 |
| 35859_f_at | PMS2L9 | U38979 | 0.498 | 0.791 |
| 40487_at | TRIM44 | W26634 | 2.104 | 1.282 |
| 1362_s_at | RXRB | M84820 | 2.483 | 1.354 |
| 33125_at | VRK2 | AL043470 | 0.386 | 0.729 |
| 35339_at | MEL | AI743606 | 2.09 | 1.275 |
| 2064_g_at | ERCC5 | L20046 | 0.485 | 0.788 |
| 32647_at | VTI1B | AF060902 | 0.419 | 0.76 |
| 33753_at | DAAM1 | AB014566 | 0.413 | 0.757 |
| 34570_at | RPS27A | S79522 | 0.461 | 0.784 |
| 41170_at | KIAA0663 | AB014563 | 0.475 | 0.792 |
| 38098_at | LPIN1 | D80010 | 0.458 | 0.787 |
| 931_at | EBI2 | L08177 | 0.486 | 0.804 |
| 33121_g_at | RGS10 | AF045229 | 0.37 | 0.743 |
| 32330_at | RPS11 | X06617 | 0.472 | 0.803 |
| 35218_at | PDCD10 | AF022385 | 0.456 | 0.795 |
| 40113_at | GS3955 | D87119 | 0.498 | 0.816 |
| 37036_at | MDN1 | AB002299 | 0.314 | 0.715 |
| 37902_at | CRYZ | L13278 | 0.362 | 0.749 |
| 1116_at | CD19 | M28170 | 1.236 | 2.121 |
| 32219_at | TLK1 | D50927 | 0.449 | 0.799 |
| 35712_at | LRRN3 | AC004142 | 0.496 | 0.823 |
| 40916_at | FLJ10097 | AL035494 | 0.448 | 0.801 |
| 37673_at | NSMAF | X96586 | 0.483 | 0.818 |
| 32792_at | P29 | AL031432 | 0.813 | 0.472 |
| 32789_at | NCBP2 | AA149428 | 0.398 | 0.776 |
| 33230_at | NMP200 | AJ131186 | 2.099 | 1.225 |
| 1764_s_at | MAZ | D85131 | 2.592 | 1.296 |
| 33305_at | SERPINB1 | M93056 | 0.454 | 0.807 |
| 259_s_at | TNF | M16441 | 2.044 | 1.214 |
| 1815_g_at | TGFBR2 | D50683 | 0.802 | 0.442 |
| 33989_f_at | TEGT | W28869 | 2.637 | 1.299 |
| 40019_at | EVI2B | M60830 | 0.44 | 0.802 |
| 34391_at | IGBP1 | Y08915 | 0.472 | 0.82 |
| 38036_at | RPIA | L35035 | 0.488 | 0.831 |
| 37670_at | ANXA7 | J04543 | 0.349 | 0.766 |
| 37999_at | CPO | D16611 | 0.444 | 0.815 |
| 35808_at |  | AL031681 | 0.45 | 0.82 |
| 38147_at | SH2D1A | AL023657 | 0.492 | 0.839 |
| 40427_at | COX17 | AA149486 | 0.466 | 0.829 |
| 40066_at | UBE1C | AF046024 | 0.454 | 0.827 |
| 1924_at | CCNH | U11791 | 0.334 | 0.771 |
| 33676_at | RPL31 | X15940 | 0.428 | 0.819 |
| 38016_at | HNRPD | M94630 | 0.48 | 0.846 |
| 35367_at | LGALS3 | AB006780 | 0.456 | 0.837 |
| 36221_at | ACYP2 | X84195 | 0.842 | 0.468 |
| 32034_at | ZNF217 | AF041259 | 0.49 | 0.851 |
| 39739_at | NACA | AF054187 | 0.481 | 0.849 |
| 39885_at | HSA9761 | W87858 | 0.804 | 0.372 |
| 32051_at | MGC2840 | AJ224875 | 0.456 | 0.843 |
| 36062_at | LPXN | AF062075 | 0.498 | 0.861 |
| 38967_at | C14orf2 | AF054175 | 0.381 | 0.814 |
| 1131_at | MAP2K2 | L11285 | 2.149 | 1.177 |
| 35795_at | HDAC6 | AJ011972 | 0.461 | 0.851 |
| 34085_at | RPL38 | Z26876 | 0.433 | 0.84 |
| 37308_at | GPR107 | AI888084 | 0.369 | 0.813 |
| 975_at | STK18 | Y13115 | 0.857 | 0.472 |
| 41372_at | KIAA0831 | AB020638 | 0.488 | 0.869 |
| 771_s_at | CD7 | D00749 | 2.111 | 1.157 |
| 36224_g_at |  | AI827895 | 0.363 | 0.826 |
| 36827_at | GOCAP1 | AF020762 | 0.453 | 0.863 |
| 41129_at | KIAA0033 | D26067 | 0.43 | 0.856 |
| 37000_at | DKFZP564B167 | AL035304 | 0.455 | 0.867 |
| 1158_s_at | CALM3 | J04046 | 2.988 | 1.219 |
| 36514_at | CGR19 | U66469 | 0.352 | 0.834 |
| 2009_at | PTK2B | U33284 | 2.579 | 1.177 |
| 41621_i_at | ZNF266 | AA868898 | 0.408 | 0.858 |
| 32193_at | PLXNC1 | AF030339 | 0.498 | 0.889 |
| 40519_at | PTPRC | Y00638 | 0.889 | 0.498 |
| 32394_s_at | RPL23 | X55954 | 0.274 | 0.804 |
| 326_i_at |  |  | 0.446 | 0.876 |
| 32248_at | PRO2730 | AL045811 | 0.473 | 0.887 |
| 40668_s_at | CD6 | U34624 | 2.47 | 1.152 |
| 34736_at | CCNB1 | M25753 | 0.384 | 0.861 |
| 39427_at | UQCRB | T79616 | 0.437 | 0.882 |
| 37313_at |  | U80017 | 0.348 | 0.858 |
| 38824_at | HTATIP2 | AF039103 | 0.438 | 0.89 |
| 34323_at | TRIP15 | AF084260 | 0.247 | 0.825 |
| 33369_at | SC4MOL | AI535653 | 0.411 | 0.888 |
| 41565_at | A2LP | AF034373 | 2.613 | 1.129 |
| 41386_i_at | KIAA0346 | AB002344 | 2.201 | 1.103 |
| 38096_f_at | HLA-DPB1 | M83664 | 0.45 | 0.909 |
| 39434_at | KIAA0592 | AB011164 | 0.481 | 0.919 |
| 834_at | ZNFN1A1 | U40462 | 2.979 | 1.134 |
| 41462_at | SNX2 | AF065482 | 0.469 | 0.922 |
| 32233_at | TOR1B | AF007872 | 1.086 | 2.16 |
| 39343_at | HSU53209 | AW026656 | 2.429 | 1.097 |
| 33818_at | VCP | AC004472 | 2.002 | 1.068 |
| 1253_at | GSK3B | L33801 | 3.062 | 1.098 |
| 36176_at | TBCC | U61234 | 0.491 | 0.944 |
| 40165_at | SE20-4 | AB015345 | 2.267 | 1.067 |
| 35985_at | AKAP2 | AB023137 | 3.716 | 1.096 |
| 37111_g_at |  | AB012229 | 4.178 | 1.089 |
| 35139_at | LOC57209 | AL049341 | 0.456 | 0.958 |
| 32395_r_at | RPL23 | X55954 | 0.401 | 0.956 |
| 41836_at | CHERP | U94836 | 2.057 | 1.033 |
| 38795_s_at | UBTF | X56687 | 2.48 | 1.04 |
| 37801_at | ATP6V0A2 | AF112972 | 2.058 | 1.028 |
| 36336_s_at | KIAA0963 | AC005390 | 3.058 | 1.043 |
| 31742_at | TNFSF14 | AF064090 | 2.073 | 1.022 |
| 41591_at | HAN11 | AI652978 | 2.014 | 1.021 |
| 1146_at |  |  | 3.446 | 1.033 |
| 35303_at | INSIG1 | U96876 | 0.472 | 0.988 |
| 39450_s_at | IDS | AF050145 | 2.193 | 1.003 |
| 40552_s_at |  | AL049987 | 0.49 | 0.998 |
| 40490_at | DDX21 | U41387 | 1.001 | 0.478 |
| 40453_s_at | SFRS5 | U30826 | 0.431 | 1.004 |
| 40110_at | IDH3B | U49283 | 0.488 | 1.013 |
| 33227_at | IL10RB | AI984234 | 0.494 | 1.018 |
| 1753_s_at | RAD23A | AD000092 | 2.822 | 0.968 |
| 40045_g_at | C18orf1 | AF009425 | 0.455 | 1.031 |
| 35934_at | EIF2S3 | L19161 | 2.037 | 0.971 |
| 32556_at | U2AF65 | X64044 | 2.333 | 0.965 |
| 40960_at | B4GALT1 | D29805 | 0.455 | 1.047 |
| 1118_at | PTGER4 | L28175 | 2.594 | 0.938 |
| 38687_at | DKFZP566D193 | AL050051 | 0.363 | 1.074 |
| 2037_s_at | RPS6KB1 | M60725 | 1.054 | 0.478 |
| 33267_at |  | AF035315 | 0.5 | 1.053 |
| 33381_at | NCOA3 | AF012108 | 2.157 | 0.944 |
| 2049_s_at | JUNB | M29039 | 2.324 | 0.928 |
| 446_at | CSNK1G2 | U89896 | 2.021 | 0.938 |
| 40495_at | CBCIP2 | AA306076 | 2.584 | 0.906 |
| 33232_at | CRIP1 | AI017574 | 0.484 | 1.081 |
| 1920_s_at | CCNG1 | X77794 | 1.122 | 0.468 |
| 38757_at | PDAP1 | U41745 | 2.265 | 0.873 |
| 34279_at | MGC8902 | AL050141 | 3.77 | 0.781 |
| 39330_s_at | ACTN1 | M95178 | 2.075 | 0.856 |
| 981_at | MCM4 | X74794 | 1.18 | 0.487 |
| 33420_g_at | API5 | U83857 | 0.476 | 1.2 |
| 37491_at | TAF1 | D90359 | 2.322 | 0.777 |
| 40592_at | IDS | L13329 | 2.03 | 0.8 |
| AFFX-HUMTFRR | TFRC | M11507 | 4.744 | 0.608 |
| 39969_at | HIST1H4C | AA255502 | 3.482 | 0.651 |
| 37506_at | FNBP3 | Z78308 | 1.276 | 0.495 |
| 274_at | ZNF148 | L04282 | 2.285 | 0.673 |
| 40215_at | UGCG | D50840 | 2.209 | 0.636 |
| 41819_at | FYB | AF001862 | 1.772 | 0.466 |
| AFFX-HUMISGF3A | STAT1 | M97935 | 2.106 | 0.549 |
